# Comprehensive Analysis of TCR and BCR Repertoires: Insights into Methodologies, Challenges, and Applications

**DOI:** 10.1186/s44342-024-00034-z

**Published:** 2025-02-24

**Authors:** Kayoung Seo, Jung Kyoon Choi

**Affiliations:** 1 Department of Bio and Brain Engineering, KAIST, Daejeon, Republic of Korea; 2 SCL-KAIST Institute of Translational Research, Daejeon, Republic of Korea

**Keywords:** T cell receptors, B cell receptors, Immune repertoire

## Abstract

The diversity of T-cell receptors (TCRs) and B-cell receptors (BCRs) underpins the adaptive immune system’s ability to recognize and respond to a wide array of antigens. Recent advancements in RNA sequencing have expanded its application beyond transcriptomics to include the analysis of immune repertoires, enabling the exploration of TCR and BCR sequences across various physiological and pathological contexts. This review highlights key methodologies and considerations for TCR and BCR repertoire analysis, focusing on the technical aspects of receptor sequence extraction, data processing, and clonotype identification. We compare the use of bulk and single-cell sequencing, discuss computational tools and pipelines, and evaluate the implications of examining specific receptor regions such as CDR3. By integrating immunology, bioinformatics, and clinical research, immune repertoire analysis provides valuable insights into immune function, therapeutic responses, and precision medicine approaches, advancing our understanding of health and disease.

## Introduction

The remarkable complexity and adaptability of the immune system are primarily driven by the vast diversity of T-cell receptors (TCRs) and B-cell receptors (BCRs) [1]. These receptors enable the immune system to recognize and respond to a wide array of antigens. The specificity of the adaptive immune response is orchestrated through the unique sequences of TCRs and BCRs, which are generated via somatic recombination processes [2]. An understanding of the immune repertoire—that is, the diversity and composition of TCRs and BCRs within an individual—provides critical insights into immune function, disease mechanisms, and therapeutic responses [3].

RNA sequencing (RNA-seq) has historically been employed to profile gene expression across heterogeneous cell populations, thereby providing a comprehensive view of transcriptomic landscapes. However, recent advancements have extended the utility of RNA-seq data to include the analysis of TCR and BCR sequences, thus enabling the exploration of immune repertoires in various physiological and pathological contexts [4]. This approach leverages the high-throughput nature of RNA-seq to capture and sequence the variable regions of TCRs and BCRs, facilitating a detailed examination of the immune landscape.

The extraction and analysis of TCR and BCR sequences from RNA-seq data necessitate the consideration of several methodological issues and the usage of sophisticated computational techniques [4, 5]. The process typically encompasses the identification of receptor-specific transcripts, the alignment and assembly of sequencing reads, and the subsequent annotation and quantification of clonotypes. These steps require the employment of specialized bioinformatics tools and pipelines that are capable of handling the complexity of receptor repertoires and ensuring the accurate representation of clonal diversity.

The analysis of immune repertoires derived from RNA-seq data presents a multitude of advantages, including the capacity to examine immune responses across a diverse array of tissues and conditions without the necessity for specialized TCR/BCR sequencing assays. This method is especially advantageous in clinical environments where there are limitations on the available sample material or where comprehensive immune profiling is required to guide treatment decisions.

The advent of next-generation sequencing has facilitated the high-coverage sequencing of TCR and BCR repertoires [6]. This has enabled the detection of rare clones with high sensitivity, as well as the identification of full-length paired chains. This capability is highly optimized for tasks such as identifying specific clones and analyzing vaccine responses [7].

This review aims to provide a comprehensive overview of the methodologies of immune repertoire analysis. The technical aspects of TCR and BCR sequence extraction, the bioinformatics pipelines employed, and the possible applications of repertoire diversity in biological studies will be discussed. By highlighting recent advancements and key findings in the field, the potential of immune repertoire analysis as a powerful tool for immunological research and clinical diagnostics will be elucidated.

## Structural and functional differences between TCR and BCR repertoires

TCR and BCR repertoires represent the diverse collections of antigen-specific receptors expressed by T cells and B cells, respectively. While both play crucial roles in adaptive immunity, they differ significantly in structure, function, and the mechanisms underlying their diversity [8, 9].

TCRs are composed of two polypeptide chains, either *α* and *β* (in most T cells) or *γ* and *δ*(in a minority of T cells), and their primary function is to recognize peptide antigens presented by major histocompatibility complex (MHC) molecules [10]. The diversity of the TCR repertoire is predominantly concentrated in the complementarity-determining region 3 (CDR3), which is generated through the recombination of TRBV, TRBD, and TRBJ segments, along with N-nucleotide insertions. This structure allows TCRs to specifically recognize processed antigens presented by MHC molecules.

In contrast, BCRs share the same basic structure as antibodies, comprising two heavy chains and two light chains. Unlike TCRs, BCRs can recognize native, unprocessed antigens directly, including proteins, carbohydrates, and lipids [11]. The diversity of the BCR repertoire arises from V(D)J recombination, similar to TCRs, but is further enhanced through somatic hypermutation and class-switch recombination during B-cell maturation [12].

TCRs are specialized for antigen recognition in the context of MHC molecules and are primarily involved in cellular immunity. They mediate the activation of T cells, which orchestrate immune responses through cytokine production and cytotoxic activity [13]. BCRs, on the other hand, function as both antigen receptors and effector molecules, as their secreted form, antibodies, plays a central role in humoral immunity by neutralizing pathogens and facilitating their clearance [14].

Both TCR and BCR repertoires rely on V(D)J recombination to generate diversity; however, the mechanisms differ in scope and outcome [2]. In TCRs, diversity is largely confined to the recombination process and the addition of N-nucleotides. In contrast, BCRs undergo additional diversification through somatic hypermutation, which introduces point mutations in the variable region to enhance antigen affinity, and class-switch recombination, which alters the antibody isotype to optimize immune responses [12].

The differences between TCR and BCR repertoires have significant implications for immune repertoire studies. TCR repertoire analysis focuses on understanding T-cell-mediated immunity, such as antigen-specific responses in infection or cancer [15]. Conversely, BCR repertoire analysis is often aimed at identifying antibodies with high antigen affinity, understanding humoral immunity, or developing therapeutic antibodies [16]. These differences highlight the distinctive roles of TCRs and BCRs in adaptive immunity and their complementary functions in protecting the host.

## Template selection in immune repertoire analysis

In conducting a repertoire analysis, the selection of the initial template is one of the most critical decisions. The template type defines the scope, sensitivity, and interpretability of the resulting repertoire data. Therefore, it is crucial to carefully choose the template, considering both the specific objectives of the study and any technical constraints that may be present.

Genomic DNA (gDNA) is a commonly utilized template in immune repertoire studies due to its stability and capacity to capture both productive and nonproductive TCR or BCR rearrangements [17]. This makes gDNA particularly suitable for estimating the total diversity of the immune repertoire, including clonotypes that are not actively expressed [18]. Since a single template is assigned to each cell, it is ideal for clone quantification, allowing for the analysis of relative abundance of clonotypes [18]. However, gDNA-based approaches do not provide information on transcriptional activity and may not reflect functional immune responses [19].

In contrast, RNA templates, specifically composed of messenger RNA (mRNA), provide a direct representation of the actively expressed repertoire [20]. mRNA-based analysis focuses on functional clonotypes, making it an optimal choice for studies aiming to understand the immune system’s dynamic responses [21]. Despite its advantages, RNA is less stable than gDNA and prone to biases during extraction and reverse transcription, which can affect the accuracy of downstream analyses [22]. Nevertheless, with the rising prevalence of single-cell RNA sequencing, concerns about potential errors and inaccuracy have decreased, and it is even possible to accurately identify rare mutations [23].

Complementary DNA (cDNA), synthesized from mRNA, serves as a common template for high-throughput sequencing [10]. It retains the functional relevance of mRNA while offering improved stability for experimental workflows [24]. However, it is subject to the same transcriptional biases as mRNA-based methods.

The selection of gDNA, RNA, or cDNA templates should be guided by the specific objectives of the repertoire analysis, such as whether the focus is on total diversity or functional clonotypes, Additionally, practical considerations, such as the quality and availability of the sample, should be taken into account.

## CDR3 only vs. full-length sequencing

In immune repertoire analysis, another key decision is whether to focus on the CDR3 region alone or to include the complete full-length sequence of the TCR or BCR chains, encompassing CDR1, CDR2, and constant regions. These two approaches differ considerably in terms of their applications, advantages, and limitations.

Using only the CDR3 region is common because it is the most variable and antigen-specific part of the receptor [18]. The CDR3 directly interacts with antigens and is primarily responsible for the diversity and specificity of immune recognition. By focusing only the CDR3 region, researchers can efficiently profile clonotypes, analyze diversity, and infer immune dynamics with reduced sequencing costs and simpler bioinformatics pipelines. However, this approach has limitations in functional interpretation. Without the surrounding regions, such as CDR1 and CDR2, which interact with the MHC molecule, it is challenging to fully understand the structural and functional aspects of antigen recognition [25, 26]. Moreover, focusing only on CDR3 limits insights into the chain pairing of TCRs (e.g., *α*- and *β*-chains), which is crucial for understanding receptor specificity [10].

In contrast, full-length sequences include additional information from the variable (V), joining (J), and constant (C) regions, along with CDR1 and CDR2. This broader context allows for a deeper understanding of receptor functionality, including MHC-binding and the overall structural conformation of the receptor [25, 26]. Full-length data also enable pairing analyses of TCR *α*- and *β*-chains or BCR heavy and light chains, which is critical for studying antigen specificity and receptor-ligand interactions [18]. All of these comprehensive analyses facilitate receptor cloning, which is also a pivotal aspect of antibody and T-cell therapy research. However, this approach comes with increased complexity in data analysis, higher sequencing costs, and potentially lower read coverage per clonotype due to the increased sequence length.

While CDR3-only sequencing is suitable for studies focused on repertoire diversity and clonal expansions, full-length sequencing provides a more comprehensive view of immune receptor function and specificity. The choice between these approaches should align with the specific goals of the study, balancing the trade-offs between scope of analysis and practical constraints such as cost and data complexity.

## Bulk sequencing vs. single-cell sequencing

Bulk sequencing is a process whereby RNA or DNA from a population of cells is pooled and then sequenced collectively. This approach provides an overview of the repertoire, capturing the diversity of clonotypes present in the sample. It is highly scalable and cost-effective, thereby enabling large-scale profiling [10]. Additionally, the workflow for bulk sequencing is relatively straightforward, and the data analysis is less computationally intensive compared to single-cell methods. However, bulk sequencing does not preserve information about receptor chain pairing or the cellular context [27]. Consequently, it averages out the repertoire at the population level, making it challenging to study individual cells or elucidate functional or phenotypic properties of specific clonotypes.

Single-cell sequencing, on the other hand, isolates individual cells for sequencing, retaining information about chain pairing and cellular context. This method is particularly useful for understanding receptor functionality, as it identifies the pairing of TCR *α*- and *β*-chains or BCR heavy and light chains, which is critical for studying antigen specificity [28]. Furthermore, single-cell sequencing can be combined with transcriptomic analysis, allowing researchers to correlate repertoire data with gene expression profiles and immune cell states [29–31]. This capability makes it a powerful tool for exploring immune cell heterogeneity and understanding the functional roles of specific clonotypes [32, 33]. However, single-cell sequencing is more costly and technically demanding, with lower throughput and potentially reduced sensitivity due to the limited number of cells analyzed [10].

The advantages of both approaches can be integrated to conduct research. Initially, an analysis can be performed at the population level through bulk sequencing, and then the study can be expanded to the single-cell level to investigate the binding affinity or expression of specific selected targets.

## Data pre-processing for repertoire analysis

**Fig. 1:**
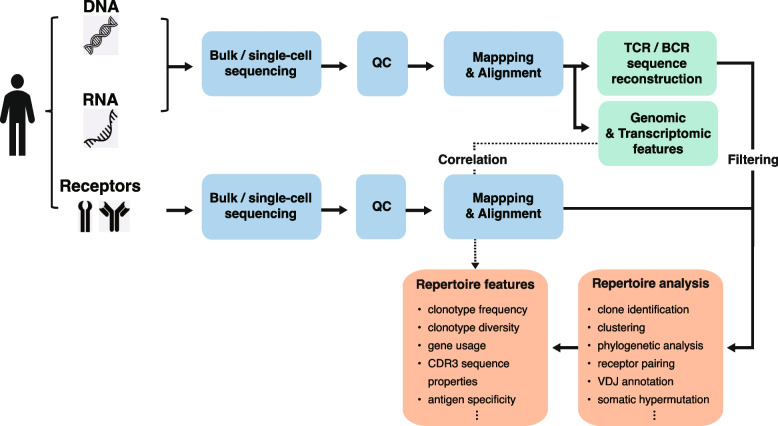
Data processing pipeline for the analysis of immune repertoires

Data pre-processing is an important stage in the analysis of immune repertoires, as it guarantees the accuracy, quality, and reliability of the sequencing data. The process consists of several steps, each of which is designed to address the particular challenges associated with immune repertoire sequencing. These steps include data quality control, alignment, and the identification of clonotypes (Fig. 1).

### Raw data quality control

The first step in data pre-processing is the removal of low quality or erroneous reads. This includes trimming adapter sequences, filtering out reads that are too short or of low quality, and eliminating reads with high error rates [12, 34]. The most commonly used quality control metric is the Phred score, which calculates the accuracy of each sequencing read and only selects reads that exceed a given threshold for further analysis [35]. It is also important to exclude sequences that do not contain the expected V(D)J gene segments or do not align with the immune receptor loci to ensure the relevance of the data to the study.

### Alignment and mapping

Following quality control, the next step is to align the reads to reference databases or gene libraries specific to TCR or BCR genes. Various bioinformatics tools, such as IMGT/HighV-QUEST [36] for BCRs or MiTCR [37] for TCRs, are commonly used to map reads to the corresponding V(D)J gene segments. This alignment process helps identify the precise gene usage (V, D, J), as well as any insertions or deletions resulting from the recombination process. Alternatively, tools such as TRUST4 [4] or MiXCR [5] can extract TCR and BCR sequences directly from pre-aligned BAM files.

### Clonotype identification and quantification

Once the reads have been mapped to the appropriate gene segments, the next step is to identify clonotypes, which are defined as unique sequences derived from the same original immune cell [38]. This involves grouping sequences that share the same CDR3 (complementarity-determining region 3) sequence, as this region is primarily responsible for antigen specificity and is highly variable [39, 40]. Clonotype identification also involves quantifying the abundance of each clonotype, which is critical for analyzing clonal expansion or contraction in response to immune challenges [41, 42]. It is important to account for sequencing errors, such as single-nucleotide polymorphisms (SNPs), which can introduce false diversity within clonotypes.

### Additional steps

For TCR and BCR analysis, particularly in studies that require an understanding of receptor pairing, the pre-processing pipeline must include steps to pair the sequences of their chains [28]. This can be challenging due to the complex nature of receptor rearrangements and the need to accurately match the α/β chains in TCRs or the heavy/light chains in BCRs. Tools like VDJtools [43] are used to link the sequences of the two chains based on their shared molecular features or cell barcodes, especially in single-cell sequencing data.

## Tools for repertoire analysis

A variety of tools are widely used in TCR and BCR repertoire analysis, ranging from those useful for raw data processing, as mentioned earlier, to more advanced tools that enable integrative analysis and visualization. Among the numerous tools, several representative ones provide distinct advantages, depending on the specific requirements of the study (Table 1).

**Table 1:** Repertoire analysis tools

Tool	Template	Strength	Platform	Language
Alakazam	TCR, BCR	Physicochemical property analysis	Bulk, single cell	R
bcRep	BCR	Inter-sample analysis of BCR	Bulk, single cell	R
Benisse	BCR	BCR repertoire embedding	Single cell	Python, R
Dandelion	TCR, BCR	Comprehensive single-cell analysis	Single cell	Python
IgBLAST	TCR, BCR	Gene annotation	Bulk, single cell	Java
IMGT/HighV-QUEST	TCR, BCR	Sequence analysis	Bulk, single cell	Java
Immcantation	TCR, BCR	Lineage construction	Bulk, single cell	Python, R
Immunarch	TCR, BCR	Visualization of features	Bulk, single cell	R
MiXCR	TCR, BCR	Comprehensive end-to-end analysis	Bulk, single cell	Java
TCRdist	TCR	Clonotype clustering	Bulk, single cell	Python
TCRMatch	TCR	Receptor specificify	Bulk, single cell	Python
Tessa	TCR	TCR repertoire embedding	Single cell	Python, R
TRUST4	TCR, BCR	Immune repertoire reconstruction	Bulk, single cell	Python
VDJtools	TCR, BCR	Clonotype identification	Bulk, single cell	Java

MiXCR [5] is a comprehensive tool designed for the rapid analysis of TCR and BCR repertoire data. It offers a complete pipeline for processing high-throughput sequencing data, including quality control, read alignment, V(D)J reconstruction, clonotype identification, and quantification. MiXCR [5] is capable of handling both RNA-seq and DNA-seq data, making it versatile for use in various experimental setups. One of its notable features is its ability to process data from both TCR and BCR repertoires in a unified framework, enabling cross-platform analysis. The advanced algorithms for V(D)J assembly of MiXCR [5] are particularly well-suited for high-throughput datasets, ensuring accurate identification of clonotypes even in the presence of sequencing errors. Additionally, it provides robust visualization tools to explore clonotype distributions and diversity metrics, facilitating downstream analysis.

Another commonly utilized tool with a focus on repertoire analysis is TRUST4 [4]. TRUST4 [4] is designed to be applicable to both bulk and single-cell RNA-seq data, and it employs a sophisticated algorithm to reconstruct TCR and BCR sequences from RNA-seq reads. One of the key strengths of TRUST4 [4] is its ability to process complex datasets with high error rates, commonly encountered in next-generation sequencing. It incorporates error-correction strategies that enhance the accuracy of clonotype identification. The distinguishing feature of TRUST4 [4] is not limited to read mapping; it also enables de novo assembly. As a result, it is capable of accurately aligning reads that have undergone recombination. TRUST4 [4] also allows for the identification of receptor sequences at a single-cell resolution when paired with single-cell RNA-seq data, offering valuable insights into T-cell and B-cell heterogeneity and functional diversity.

IMGT/HighV-QUEST [36] is a highly specialized tool for the analysis of BCR and TCR sequences based on the IMGT (international ImMunoGeneTics) database, which is one of the most comprehensive repositories for immune receptor genes. It performs a detailed V(D)J gene alignment, providing users with information on the gene usage and potential mutations in the CDR regions and is optimized for identifying clonotypes based on both DNA and RNA sequences. The tool also offers a high degree of customization, allowing users to specify certain parameters for alignment and analysis, making it suitable for a variety of experimental designs.

IgBlast [44], developed by the National Center for Biotechnology Information (NCBI), is a tool designed for high-speed and accurate alignment of immunoglobulin and TCR sequences to the IMGT reference database. While IgBlast [44] does not perform full repertoire analysis, it is often used in conjunction with other tools like IMGT/HighV-QUEST [36] for more in-depth BCR repertoire characterization.

VDJtools [43] is a flexible tool designed for the post-processing, visualization, and analysis of immune repertoire sequencing data. It is especially useful for comparing and visualizing repertoire data across different conditions, such as between healthy and diseased samples. The VDJtools [43] enables the calculation of a range of diversity metrics, including Shannon entropy, Simpson’s index, and clonal richness, which are essential for elucidating the diversity of immune repertoires. It also provides algorithms for statistical analysis, helping researchers assess the significance of changes in repertoire composition. Moreover, VDJtools [43] supports integration with other platforms, such as MiXCR [5] and TRUST4 [4], enabling users to combine different tools within a single analytical framework.

In summary, tools like MiXCR [5] and TRUST4 [4] are suitable for comprehensive analysis workflows including nearly all fundamental analysis, while IMGT/HighV-QUEST [36] and IgBlast [44] offer detailed gene alignment and annotation. VDJtools [43] excels in post-processing and visualization, and specialized tools like TCRdist [45, 46] and TCRmatch [47] provide advanced clustering and pairing functionalities. The most suitable tool should be selected and used based on the specific objectives of the analysis.

## Extractable feature types

The analysis of immune repertoires offers valuable insights into the diversity and responsiveness of the immune system to various stimuli, including infections, tumors, and autoimmune conditions [23]. By examining the sequencing data of TCR and BCR repertoires, several key features can be extracted, each providing important information about the immunological landscape of a sample (Table 2). These features are crucial for understanding immune dynamics, assessing disease states, and guiding therapeutic strategies.

**Table 2:** Features extractable from immune repertoire

Feature type	Feature description	Tools
Clonotype diversity	Number of clonotypes	VDJtools, bcRep, Dandelion, Immunarch
	Number of clusters	TCRdist, RIMA, Immcantation
	Shannon entropy	VDJtools, RIMA, bcRep
	Simpson’s index	VDJtools, Alakazam, bcRep, Immunarch
	Jaccard index	VDJtools
	Gini coefficient	bcRep, Dandelion, Immunarch
	Pielou’s index	VDJtools, RIMA
	Richness	Alakazam, bcRep, Immunarch
Chemical properties of sequences	Length	IMGT/HighV-QUEST, IgBLAST, VDJtools, bcRep, …
	Nucleotide composition	Alakazam, bcRep
	Gravy index	Alakazam
	Bulkiness of amino acids	Alakazam
	Polarity of amino acids	Alakazam
	Net charge	Alakazam
	Fraction of basic amino acids	Alakazam
	Fraction of acidic amino acids	Alakazam
	Fraction of aromatic amino acids	Alakazam
	Aliphatic index	Alakazam
Clonotype frequency		TRUST4, MiXCR, Immunarch
Clonotype distances		Benisse, Immunarch, Immcantation, TCRdist
V(D)J gene usage		IMGT/HighV-QUEST, TCRdist, bcRep, Immunarch, …
Somatic hypermutation rate		IgBLAST, bcRep, Immcantation, MiXCR
Receptor specificity		TCRMatch
Repertoire similarity		VDJtools
Clone functionality		IMGT/HighV-QUEST, bcRep

One of the most fundamental characteristics that can be derived from TCR and BCR repertoire analysis is clonotype diversity. This term refers to the variety of unique receptor sequences present within the repertoire, which are typically characterized by the diversity of the CDR3 regions [39, 40]. Clonotype diversity is indicative of the magnitude of the immune response and can be evaluated using metrics such as Shannon entropy or Simpson’s index. VDJtools [43] can calculate various diversity metrics, providing a comprehensive picture of repertoire diversity. High diversity is frequently associated with a robust immune system capable of recognizing a diverse array of pathogens, whereas reduced diversity may indicate immunodeficiency or an oligoclonal expansion, such as observed in certain cancers [48, 49] or autoimmune diseases [50].

The analysis of clonotype frequency within the repertoire can reveal whether there has been a clonal expansion or contraction, which in turn reflects the immune system’s response to specific antigens [41]. VDJtools [43] allows for the clustering of clonotypes, which can reveal patterns of clonal expansion or contraction and is also capable of visualizing repertoire distributions. Clonal expansion occurs when certain clones proliferate in response to infection, vaccination, or tumor progression [41]. This can be quantified by the relative abundance of specific clonotypes in the sample. In contrast, clonal contraction refers to the decrease in the frequency of particular clones over time, often following the resolution of an immune challenge. The monitoring of these dynamics provides valuable insights into the kinetics of immune responses and immune memory formation [51].

TCR and BCR repertoire analysis also involves the examination of repertoire composition, particularly the usage of specific V, D, and J gene segments. This feature provides insight into the manner in which the immune system assembles its receptors and can reveal biases in gene usage. For example, certain diseases or immune responses may be associated with preferential use of specific V genes, which can provide clues about the underlying mechanisms of immune recognition [52, 53]. In BCR repertoires, the analysis of the usage of heavy and light chain variable regions can also assist in the identification of patterns related to antigen specificity or affinity maturation [54]. IMGT/HighV-QUEST [36] is especially effective for detailed gene usage analysis, as it relies on the comprehensive IMGT database, which includes a large collection of annotated V, D, J, and C region sequences. It enables the identification of the V, D, and J genes used in receptor formation and can provide insights into gene segment preferences within a sample.

The length and sequence composition of the CDR3 region represent another important feature derived from immune repertoire analysis. CDR3 length can influence receptor specificity and is often used as a marker to differentiate between diverse TCR and BCR clones [55]. Additionally, the specific nucleotide sequence of the CDR3 region determines antigen recognition, and its analysis can provide information on the types of antigens that have been encountered by the immune system. For instance, certain CDR3 motifs or sequence patterns may be associated with particular pathogens or tumor antigens, making it a useful feature in studying disease-specific immune responses. IMGT/HighV-QUEST [36] enables examining CDR3 sequences, including their length and nucleotide composition. The chemical properties of the amino acids that comprise each CDR3 sequence can also be considered defining features of both the sequence itself and the overall repertoire. The chemical properties of each amino acid, including charge and polarity, can be calculated and aggregated at the sequence level using Alakazam [56]. Moreover, these properties can be further converged to represent each clone or the entire repertoire.

For TCRs, identifying receptor pairing is crucial for understanding antigen specificity [28]. The ability to pair α/β chains in TCRs and heavy/light chains in BCRs provides a complete insight of receptor functionality and antigen recognition. Receptor pairing data can also help identify potential cross-reactivity in antigen recognition, which may have implications for autoimmune diseases or vaccine development.

In the case of BCR repertoires, somatic hypermutation and affinity maturation are critical features. Somatic hypermutation introduces point mutations in the variable region of BCR genes during the germinal center reaction, leading to the generation of BCRs with higher affinity for antigens [57]. The extent and pattern of somatic hypermutation can provide insights into the maturation of the immune response and the effectiveness of the immune system in responding to pathogens or tumors. Analyzing this process is particularly important in understanding the development of long-lived antibody responses, such as those generated during infection or vaccination. IgBlast [44] can be applied to examine somatic hypermutation patterns and mutations in CDR regions.

By grouping similar clonotypes, repertoire clustering can be performed to identify potential antigen-driven expansions [38]. This feature enables the identification of specific clones that may be enriched in certain immune responses. Additionally, clustering can help assess the clonal overlap between different individuals or populations, offering insights into shared immune responses and the potential for immune evasion in diseases like cancer or viral infections. TCRdist [45, 46] is a tool designed for the analysis and clustering of TCR sequences based on their CDR3 sequences. The tool calculates the distance between TCR clonotypes using a variety of distance metrics, including sequence identity or structural similarity, which is valuable for clustering related TCRs that may share antigen specificity. This tool is particularly useful for investigating the similarity of TCR repertoires within and across samples, helping to identify potential antigen-driven expansions and the extent of clonal overlap. Furthermore, TRUST4 [4] also provides clustering of TCR and BCR clonotypes based on sequence similarity. The similarity of BCR clonotypes is attributed to somatic hypermutation, a process that occurs during B-cell activation. Consequently, clonotypes within the same cluster can be considered to belong to the same lineage during proliferation.

Tracking the temporal dynamics of the TCR and BCR repertoires provides valuable insights into immune memory, immune tolerance, and response durability. Longitudinal studies can reveal how the immune system adapts to new infections or vaccinations, how clonal expansions evolve, and how immune tolerance mechanisms develop over time. Repertoire analysis can thus inform the study of immune aging, autoimmunity, and chronic infections and can generate interactive visualizations of clonal relationships and lineage trees.

## Applications

**Fig. 2:**
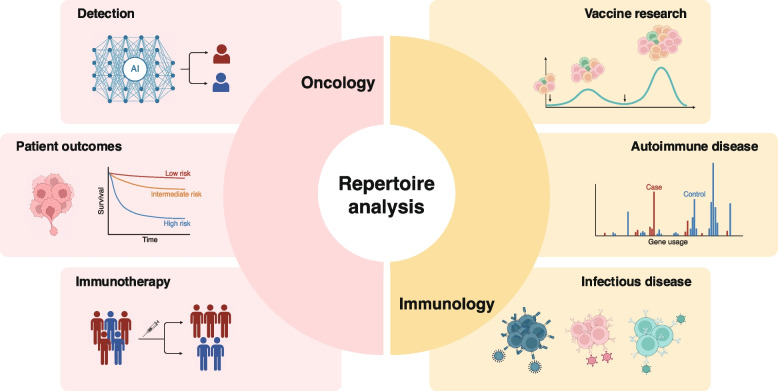
Potential applications of immune repertoire analysis

Studies utilizing TCR and BCR repertoire analyses span a wide range of applications in immunology and oncology, extending beyond the identification of disease-specific receptors (Fig. 2). The advent of the COVID-19 pandemic has given rise to a notable increase in research about vaccines. Efforts to quantify vaccine-induced immune responses have been diverse, with single-cell repertoire sequencing data providing expanded opportunities to apply a broader range of repertoire features. For instance, clonotype frequency dynamics can be analyzed to identify TCR or BCR clone expansion triggered by vaccination. Additionally, this pattern of clone expansion could potentially be predicted by associating it with features extracted from pre-vaccination DNA-seq or RNA-seq data [58].

Research aimed at identifying distinct repertoire features in autoimmune diseases is ongoing. Among numerous studies, it has been demonstrated that patients with systemic lupus erythematosus (SLE) exhibit increased TCR and BCR clonotype diversity, along with distinct gene usage patterns compared to controls [59]. Additionally, changes in the BCR repertoire features have been observed during the treatment process in patients with various immune-related diseases [60]. These findings have the potential to serve as diagnostic markers or therapeutic targets in the future.

In the field of infectious disease studies, repertoire analysis enables the identification of pathogen-specific TCR and BCR clones in a straightforward manner. By comparing the repertoire before and after infection, the expansion of specific clones can be observed, which can be utilized in the development of vaccines [61].

The features derived from the repertoire analysis described above have been quantified and integrated into inputs for machine learning models. These models can be developed to classify disease phenotypes, predict drug or therapeutic responses, or assess prognostic outcomes. In particular, the use of RNA-seq data obtained from liquid biopsies as a template offers a noninvasive and straightforward approach for repertoire analysis, enabling efficient screening for various applications.

TCR diversity can be considered a prognostic marker in melanoma patients. Specifically, melanoma patients with greater TCR evenness and richness in both blood and lymph nodes have been observed to demonstrate longer progression-free overall survival [62]. An increased diversity in the TCR repertoire of tumor-infiltrating T cells has been identified as a prognostic indicator in various cancer types [63].

Following immunotherapy administration, tracking TCR clonotype frequency dynamics or diversity can be employed as a marker to predict future immunotherapy responses. In patients with advanced melanoma, both increased richness and evenness of TCR diversity in the peripheral blood, which were previously identified as markers of a good prognosis, corresponded to greater clinical benefit following treatment with ipilimumab, a CTLA-4 inhibitor [64]. Additionally, high richness was identified as a marker of enhanced therapeutic efficacy and a better prognosis in NSCLC patients undergoing anti-PD-1 immunotherapy [65].

## Conclusion

In this review, we explored the key considerations and methodologies involved in TCR and BCR repertoire analysis, a crucial approach for understanding the adaptive immune system’s complexity and functionality. By examining the functional and structural differences between TCRs and BCRs, out objective was to elucidate the manner in which these variations influence the respective roles of these receptors in immune responses. We discussed the critical factors when choosing templates, whether DNA or RNA, and analyzed the implications of focusing on the CDR3 region versus the full-length receptor sequence. Additionally, the comparative advantages and limitations of using bulk versus single-cell sequencing data were highlighted, demonstrating how these choices impact the resolution and scope of immune repertoire studies.

Furthermore, we outlined the procedure of data processing for repertoire analysis, including quality control, alignment, and clonotype identification, as a fundamental step in ensuring robust and reliable results. A variety of computational tools were examined for their capabilities and suitability for different stages of repertoire analysis. This enables researchers to customize workflows to their specific study goals. Moreover, we explained the diverse features of a sample that can be derived from repertoire data; all of which provide valuable insights into immune dynamics and functionality.

Repertoire analysis is a discipline that bridges immunology, bioinformatics, and clinical research. It offers a powerful lens through which to investigate immune responses, discover therapeutic targets, and develop precision medicine approaches. By integrating appropriate methodologies, tools, and analytical strategies, researchers can fully leverage immune repertoire data to deepen our understanding of the immune system and its role in health and disease.

## Data Availability

No datasets were generated or analysed during the current study.
